# Immunization with a Biofilm-Disrupting Nontypeable *Haemophilus influenzae* Vaccine Antigen Did Not Alter the Gut Microbiome in Chinchillas, Unlike Oral Delivery of a Broad-Spectrum Antibiotic Commonly Used for Otitis Media

**DOI:** 10.1128/mSphere.00296-20

**Published:** 2020-04-15

**Authors:** Michael T. Bailey, Christian L. Lauber, Laura A. Novotny, Steven D. Goodman, Lauren O. Bakaletz

**Affiliations:** a Center for Microbial Pathogenesis, Abigail Wexner Research Institute at Nationwide Children’s Hospital, Columbus, Ohio, USA; b Institute for Genomic Medicine, Abigail Wexner Research Institute at Nationwide Children’s Hospital, Columbus, Ohio, USA; c Department of Pediatrics, The Ohio State University College of Medicine, Columbus, Ohio, USA; University of Kentucky

**Keywords:** DNABII, tip-chimer peptide, dmLT, otitis media, microbiome

## Abstract

The use of broad-spectrum antibiotics to treat diseases, such as the highly prevalent pediatric disease otitis media (OM), contributes significantly to the worldwide emergence of multiple-antibiotic-resistant microbes, and gut dysbiosis with diarrhea is a common adverse sequela. Moreover, for many diseases, like OM, biofilms contribute significantly to chronicity and recurrence, yet biofilm-resident bacteria are characteristically highly resistant to antibiotics. The most cost-effective way to both prevent and resolve diseases like OM, as well as begin to address the problem of growing antibiotic resistance, would be via the development of novel approaches to eradicate bacterial biofilms. Toward this goal, we designed a vaccine antigen that induces the formation of antibodies that prevent biofilm formation and, thereby, experimental OM in the middle ears of chinchillas by the predominant Gram-negative pathogen responsible for this disease, nontypeable Haemophilus influenzae. These antibodies also significantly disrupt preexisting biofilms formed by diverse pathogens. Whereas preclinical data strongly support the continued development of this vaccine antigen, which targets an essential structural element of bacterial biofilms, a concern has been whether active immunization would also lead to unintended collateral damage in the form of an altered gut microbiome. To address this concern, we assessed changes in the microbiome of the chinchilla gut over time after the delivery of either amoxicillin-clavulanate, the standard of care for OM, or after immunization with our biofilm-targeted vaccine antigen either via a traditional subcutaneous route or via a novel noninvasive transcutaneous route. We show that differences in the abundance of specific taxa were found only in the stools of antibiotic-treated animals.

**IMPORTANCE** The prevalence of chronic and recurrent diseases, combined with the overuse/abuse of antibiotics that has led to the sobering emergence of bacteria resistant to multiple antibiotics, has mandated that we develop novel approaches to better manage these diseases or, ideally, prevent them. Biofilms play a key role in the pathogenesis of chronic and recurrent bacterial diseases but are difficult, if not impossible, to eradicate with antibiotics. We developed a vaccine antigen designed to mediate biofilm disruption; however, it is also important that delivery of this vaccine does not induce collateral damage to the microbiome. The studies described here validated a vaccine approach that targets biofilms without the consequences of an altered gut microbiome. While delivery of the antibiotic most commonly given to children with ear infections did indeed alter the gut microbiome, as expected, immunization via traditional injection or by noninvasive delivery to the skin did not result in changes to the chinchilla gut microbiome.

## Abstract (precis)

The prevalence of chronic and recurrent diseases, combined with the overuse/abuse of antibiotics that has led to the sobering emergence of bacteria resistant to multiple antibiotics, has mandated that we develop novel approaches to better manage these diseases or, ideally, prevent them. Biofilms play a key role in the pathogenesis of chronic and recurrent bacterial diseases but are difficult, if not impossible, to eradicate with antibiotics. We developed a vaccine antigen designed to mediate biofilm disruption; however, it is also important that delivery of this vaccine does not induce collateral damage to the microbiome. The studies described here validated a vaccine approach that targets biofilms without the consequences of an altered gut microbiome. While delivery of the antibiotic most commonly given to children with ear infections did indeed alter the gut microbiome, as expected, immunization via traditional injection or by noninvasive delivery to the skin did not result in changes to the chinchilla gut microbiome.

## INTRODUCTION

With the sobering global rise in antibiotic-resistant bacteria, there is a tremendous need to develop better therapeutic approaches and immunization regimes to manage infectious diseases. One such disease is otitis media (OM), which is highly prevalent in the pediatric population worldwide (1, 2). The prescribing of broad-spectrum antibiotics for OM is considered a primary driving force behind the rise in antibiotic resistance not only in the three predominant bacterial species that cause OM (e.g., nontypeable Haemophilus influenzae [NTHi], Streptococcus pneumoniae, and Moraxella catarrhalis) but also in multiple additional pathogens due to the propensity of microbes to share antibiotic resistance genes through horizontal gene transfer (3, 4).

OM is actually a spectrum of clinical entities that includes acute OM, chronic OM, recurrent OM, and chronic suppurative OM, with the last one being the most severe (5). Regardless of the specific definition, OM is a complex, multifactorial, and polymicrobial disease in which highly recalcitrant biofilms contribute significantly to pathogenesis (6–10). The term “biofilm” is constantly being refined; however, in general, a biofilm is defined as a 3-dimensional bacterial community that either adheres to an abiotic or biotic surface or is present as an aggregate encased in a self-produced matrix and that has multiple properties that confer an inherently resistant-to-eradication phenotype (11, 12). Due to the importance of biofilms in >80% of all bacterial diseases (13), many laboratories are focused on the development of novel ways to either eradicate biofilms or prevent their formation (14, 15).

In our own efforts to develop a broadly effective vaccine to prevent OM, we strove to better understand the biofilms built by those bacteria that predominate in OM, with a particular focus on NTHi. As such, we have characterized the NTHi biofilm matrix, and one of our discoveries was not only that extracellular bacterial DNA (eDNA), first demonstrated in the Pseudomonas aeruginosa biofilm by Whitchurch et al. (16), is an integral part of the NTHi biofilm matrix but also that the eDNA contained within this matrix is arranged as a lattice that confers structural stability to the biofilm (17). Importantly, positioned at the junction of each pair of crossed strands of eDNA in this lattice is a linchpin protein, a member of the ubiquitous DNABII family of bacterial DNA-binding proteins (18).

The DNABII family of proteins plays an essential role in maintaining the structural integrity of bacterial biofilms (19). The DNABII family has only two members, integration host factor (IHF) and histone-like protein (HU), best known for their roles intracellularly in a range of important nucleoprotein interactions (19). Family members function as homo- or heterodimers and initiate binding to DNA primarily via insertion of the tips of highly conserved β-ribbons into the minor groove (19). We showed in earlier work that DNABII proteins also play an important role outside the bacterial cell, where they contribute significantly to the biofilm’s eDNA scaffold (18, 20). In multiple follow-up studies, we continued to define the role of the DNABII proteins in the bacterial biofilm as well as attempted to better understand their biological importance to determine if these proteins could serve as a target for the development of a novel biofilm-disrupting vaccine immunogen or therapeutic agent.

We subsequently showed that when established biofilms are incubated with antiserum directed against a DNABII protein, DNABII proteins free in the environment are bound with a high affinity by these specific antibodies, inclusive of their DNA-binding surfaces. Antibody binding to the tips of the DNABII proteins prevents the association of IHF or HU with eDNA. These events reduce the reservoir of free DNABII proteins, and this reduction in turn shifts the equilibrium away from DNABII bound to the biofilm’s eDNA scaffold and causes the subsequent rapid collapse of the biofilm structure with the release of the resident bacteria (21). These antisera effectively disrupt biofilms formed by not only the predominant pathogens of OM but also multiple additional diverse pathogens, including those formed by the high-priority, highly antibiotic-resistant ESKAPE (Enterococcus faecium, Staphylococcus aureus, Klebsiella pneumoniae, Acinetobacter baumannii, Pseudomonas aeruginosa, and *Enterobacter* species) pathogens (20, 22–26).

As the collective result of epitope mapping efforts and preclinical studies that demonstrated the protective and therapeutic potential of the DNABII-derived vaccine antigens (18, 21, 25–27), we hypothesized that this DNABII-targeted approach could have important ramifications in our efforts to develop a platform technology for better biofilm disease management and/or prevention universally. Whereas the biofilm disruption efficacy has now been demonstrated both *in vitro* (18, 21, 22, 24, 28, 29) and also in preclinical studies in three animal models of distinct human diseases (18, 21, 25–27), an important question remains: what is the potential for a biofilm-directed immunogen to also perhaps induce unwanted collateral damage in the form of alteration of either the respiratory tract or the gastrointestinal tract microbiome, given the universal role of the DNABII family in biofilm architecture, including in members of the normal, healthy microbiome?

To address this question, we compared the relative potential for gut microbiome disruption when chinchillas either were given amoxicillin-clavulanate, a first-line antibiotic for children with OM (3), or were immunized by injection (parenterally) with a peptide immunogen derived from the DNABII proteins in which known protective epitopes from the β-ribbon turns of the DNA-binding surface (tips) of both the alpha and beta subunits were colinearly synthesized with a short joining peptide segment to produce a tip-chimer peptide (27). We also comparatively tested a transcutaneous immunization (TCI) regime in which the tip-chimer peptide was delivered locally via rubbing onto the postauricular skin (i.e., the area just behind the ear), which is a proven means to induce significant protective efficacy while also intentionally limiting the induction of systemic antibody (30, 31). Immunized animals or those given antibiotics were compared to animals in the control cohorts via both histological and microbiome analyses for the relative changes to the gastrointestinal tract that were induced. We found that consistent changes to the microbiome were detected only in the stool samples recovered from animals given antibiotics and not in those recovered from animals immunized with the DNABII-directed immunogen. The differences in the effect on the microbiome between cohorts that were given antibiotic and those that were immunized are discussed.

## RESULTS

### Induction of an immune response after immunization with the tip-chimer peptide admixed with the adjuvant dmLT.

To assess whether the anti-DNABII antibodies induced by immunization (see Fig. S1 in the supplemental material) caused alterations to the gut microbiome, we first confirmed the production of immunogen-specific serum antibodies after parenteral or transcutaneous immunization. Parenteral (subcutaneous [SQ]) immunization with the tip-chimer peptide admixed with the adjuvant double mutant heat-labile toxin (dmLT) resulted in an immunogen-specific geometric mean titer of 4.2 × 10^4^ ± 2.3, whereas TCI yielded a markedly reduced serum antibody titer of 211 ± 1.5. These antibody quantities and the substantial difference observed between the two regimens were expected, as parenteral immunization induces strong serum antibody levels, whereas TCI of the chinchilla induces a smaller amount of serum antibodies, despite mediating effective antibody production locally in the middle ear (30–33). Tip-chimer-specific serum titers in chinchillas administered dmLT only were equivalent to the background titers. Thus, with the induction of a tip-chimer peptide-specific systemic immune response confirmed, the potential for immunization-induced alterations to the gut microbiome was examined.

10.1128/mSphere.00296-20.1 FIG S1 Timeline depicting the times of oral administration of antibiotic or immunization by the transcutaneous route or the parenteral regime (arrows) and indication of dates of specimen collection (triangles). Download FIG S1, TIF file, 0.10 MB. Copyright © 2020 Bailey et al. 2020 Bailey et al. This content is distributed under the terms of the Creative Commons Attribution 4.0 International license.

### 16S rRNA gene sequencing.

For fecal samples, 2,695,674 high-quality 16S rRNA gene sequences were obtained from 96 samples, with an average of 28,079 ± 5,824 sequences being obtained per sample. However, for nasopharyngeal (NP) lavage samples, only 30,791 high-quality 16S rRNA gene sequences were obtained from 81 samples, with an average 380 ± 147 sequences being obtained per sample. Data from the NP lavage samples are not presented, because the low sequencing depth and high variability in the taxa found in these samples limited our confidence in them. The sequencing depth was, however, sufficient in the fecal samples and thus warranted continuation of the diversity analyses.

### Alpha diversity.

**FIG 1:**
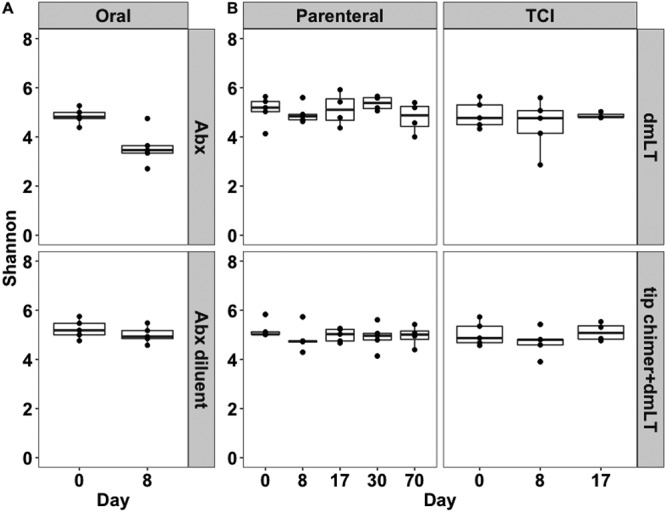
Antibiotics but not immunization affected microbial alpha diversity in the stool. The Shannon diversity index was calculated from sequences derived from stool samples. (A) The Shannon diversity index was equivalent in stool samples from animals administered Abx or diluent on study day 0 (i.e., baseline), but there was a significant reduction in the Abx-treated cohort on study day 8 compared to that at baseline (*P* < 0.05, using a repeated-measures *t* test). The Shannon diversity index did not change between days 0 and 8 in animals delivered the diluent. (B) The Shannon diversity index was not significantly different between the cohorts immunized with tip-chimer peptide–dmLT or dmLT alone on study day 0 (i.e., baseline) or on any subsequent sampling day.

We analyzed the bacterial community alpha diversity in the fecal samples from chinchilla cohorts that were given an antibiotic cocktail (Abx) or antibiotic diluent (diluent) or that were immunized by parenteral administration or via TCI with the tip-chimer peptide–dmLT or the adjuvant dmLT only (Fig. S1) to determine if any treatment induced a widespread gut microbiome alteration across time. There were no significant differences in the Shannon diversity index between any of the treatment groups at baseline (i.e., study day 0). However, using an independent samples Student's *t* test, we observed that the Shannon diversity index was significantly decreased in fecal samples from animals given Abx compared to that in animals given the diluent-only control on study day 8 (*P = *0.009) (Fig. 1A). Consistent with this finding, the alpha diversity in the Abx-treated group was significantly lower on day 8 than it was on day 0 (*P = *0.015), but alpha diversity did not change in the diluent-treated group (Fig. 1A). In chinchillas immunized with the tip-chimer peptide–dmLT (parenterally or transcutaneously), the alpha diversity was similar to the alpha diversity found in the cohort immunized with dmLT only on study days 0 (baseline), on days 8 and 17 (for parenteral and TCI administration), and on days 30 and 70 (for parenteral administration only) (Fig. 1B). Consistent with this observation, there were no differences in the dmLT or the tip-chimer–dmLT groups on study day 8, 17, 30, or 70 when the results were compared to those for their respective day 0 (baseline) time point (Fig. 1B). Thereby, delivery of amoxicillin-clavulanate was the only treatment that affected alpha diversity.

### Beta diversity.

**FIG 2:**
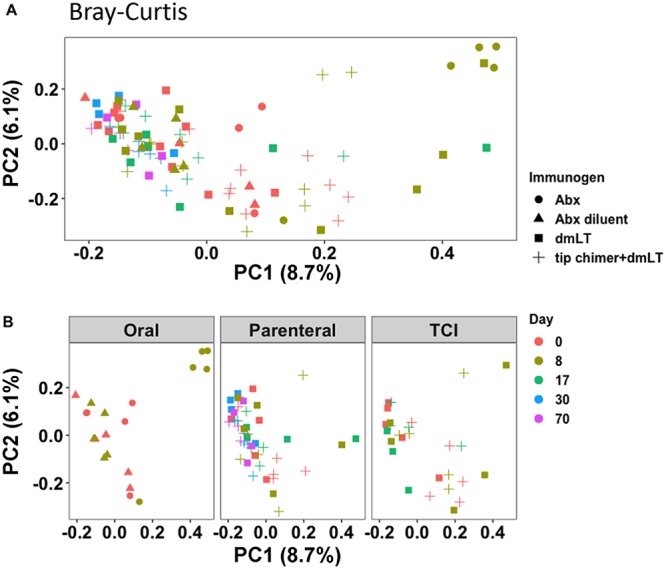
Microbial beta diversity in the stool was altered by delivery of antibiotic. (A) Principal-coordinate (PC) analysis of Bray-Curtis dissimilarities of fecal communities demonstrated that 4 out of the 5 samples from the Abx-treated cohort (along with 1 out of the 5 samples from animals administered dmLT by TCI) clustered separately from the other samples (*P = *0.029 by PerMANOVA using 999 randomizations of the data). (B) The results for samples from orally treated animals, parenterally treated animals, and animals receiving TCI are plotted separately to better illustrate the separation of the animals in the Abx-treated group on study day 8 but the lack of separation of animals in the dmLT- and tip-chimer–dmLT-treated groups on any of the sample days.

We used Bray-Curtis dissimilarity analysis and permutational multivariate analysis of variance (PerMANOVA) to monitor the changes in the overall community composition that resulted from one of the six treatments delivered. Beta diversity was similar in all cohorts at baseline; however, in concordance with the findings of previous studies using preclinical animal models, Abx significantly changed the overall beta diversity (PerMANOVA, *P* = 0.025) of the fecal communities (Fig. 2). Similar results were evident when other measures of beta diversity were assessed, including Jaccard distances as well as weighted and unweighted UniFrac distances (Fig. S2), confirming that beta diversity was different between the Abx-treated group and the Abx-diluent-treated group. This difference was due to an antibiotic-induced change in beta diversity, since the beta diversity on study day 8 was significantly different from that on day 0 (baseline) in the Abx-treated cohort (PerMANOVA, *P* = 0.029) but not in the cohort delivered diluent (Fig. 2B). Thus, differences in beta diversity were induced by administration of an antibiotic.

10.1128/mSphere.00296-20.2 FIG S2 Microbial beta diversity in the stool was affected by antibiotic administration but not by immunization. Principal-coordinate analysis of the results obtained by analysis of unweighted UniFrac (A), weighted UniFrac (C), and Jaccard (E) distances, showing that samples from 4 out of 5 samples from Abx-treated animals from study day 8 as well as 1 out of 5 samples from dmLT-treated animals from study day 8 clustered separately from all other samples (*P* < 0.05 by PerMANOVA for each distance metric). The unweighted UniFrac (B), weighted UniFrac (D), and Jaccard (F) distances from orally treated animals, parenterally immunized animals, and animals receiving TCI are plotted separately to better illustrate the separation of the Abx-treated group on study day 8 but the lack of separation in the dmLT- and tip-chimer–dmLT-treated groups on any of the sample days. Download FIG S2, TIF file, 1.0 MB. Copyright © 2020 Bailey et al. 2020 Bailey et al. This content is distributed under the terms of the Creative Commons Attribution 4.0 International license.

In contrast, the beta diversity among chinchillas immunized with the tip-chimer peptide–dmLT was similar to that in the adjuvant-only cohort on study days 0 (baseline), 8, 17, 30, and 70 (Fig. 2A). When assessed longitudinally, neither tip-chimer peptide–dmLT nor dmLT led to changes in beta diversity on study day 8, 17, 30, or 70 compared to that at baseline (Fig. 2A). The lack of effects on beta diversity was evident regardless of whether tip-chimer peptide–dmLT or dmLT alone was given parenterally or transcutaneously (Fig. 2B).

### Taxonomic abundances.

**FIG 3:**
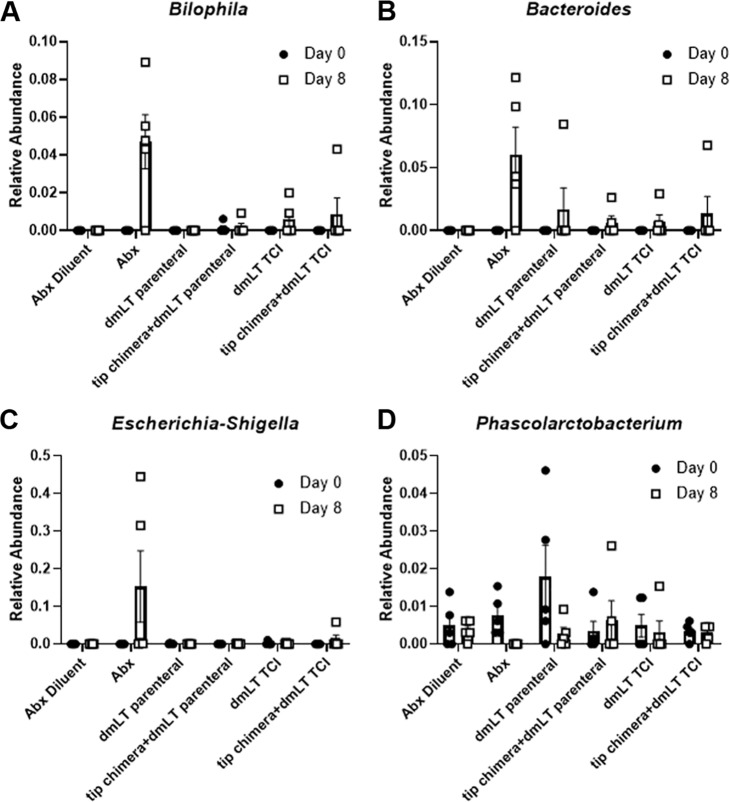
Antibiotic treatment but not immunization with a DNABII-directed immunogen affects the relative abundance of multiple bacterial taxa in fecal samples. Random forest analysis followed by Boruta feature selection indicated that samples from the Abx-treated group on study day 8 had differences in the abundances of *Bilophila* (A), *Bacteroides* (B), *Escherichia-Shigella* (C), and *Phascolarctobacterium* (D) from those in the diluent-treated group. Random forest analysis did not identify any bacterial taxa whose abundance was distinct in any of the groups immunized with tip-chimer peptide–dmLT or dmLT only via the parenteral or transcutaneous route.

As changes in both alpha and beta diversity were detected in the cohort administered antibiotic compared to that in animals immunized with the tip-chimer peptide, we next examined whether there were differences in specific bacterial taxa. Random forest (RF) analysis alongside Boruta feature selection in fecal samples collected from animals that received antibiotic revealed 4 bacterial genera whose abundance was distinct from that in samples collected from all other cohorts (Fig. 3). In particular, the abundance of *Bilophila*, *Bacteroides*, and *Escherichia-Shigella* was increased in stool samples after 8 days of antibiotic treatment, whereas the abundance of *Phascolarctobacterium* was decreased. No other treatment significantly affected bacterial abundance. Therefore, only delivery of an antibiotic significantly affected the fecal microbial community composition.

### Weight changes.

**FIG 4:**
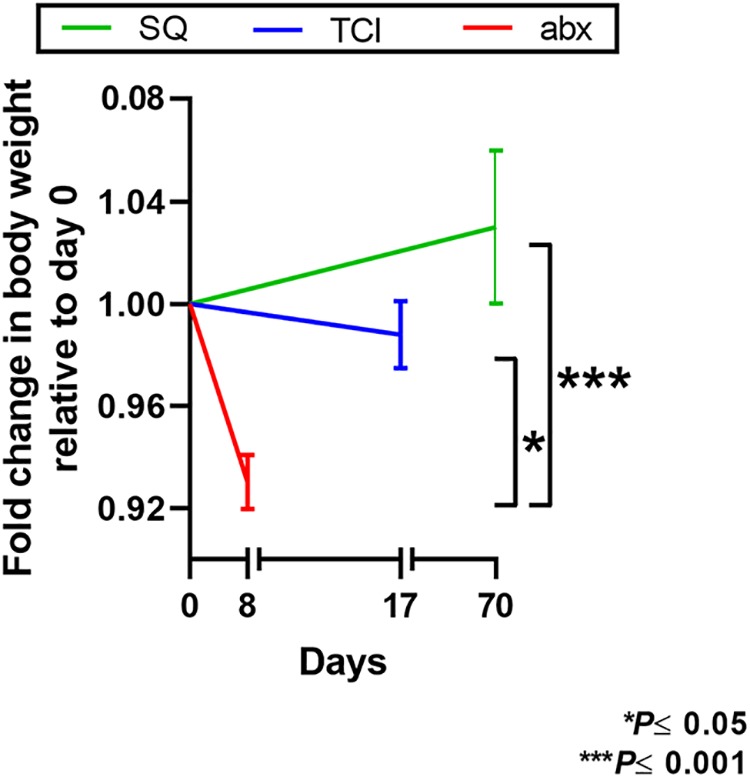
Body weight loss among the chinchilla cohorts over the course of treatment or immunization. Only the cohort that received oral antibiotics showed a statistically significant loss of weight due to treatment relative to that in the cohort that was immunized transcutaneously or the cohort that was immunized subcutaneously (*P* ≤ 0.05 and *P* ≤ 0.001, respectively).

As an additional measure of treatment or immunization effect, chinchillas were weighed frequently from the time of study initiation to the time of termination. Only those animals that received oral antibiotics demonstrated a significant loss in weight compared to the weight of the animals either in the cohort immunized via TCI (*P* ≤ 0.05) or in the cohort immunized via the SQ route (*P* ≤ 0.001) (Fig. 4). There were no other significant differences in weight loss among the three cohorts.

### Histological analyses.

**FIG 5:**
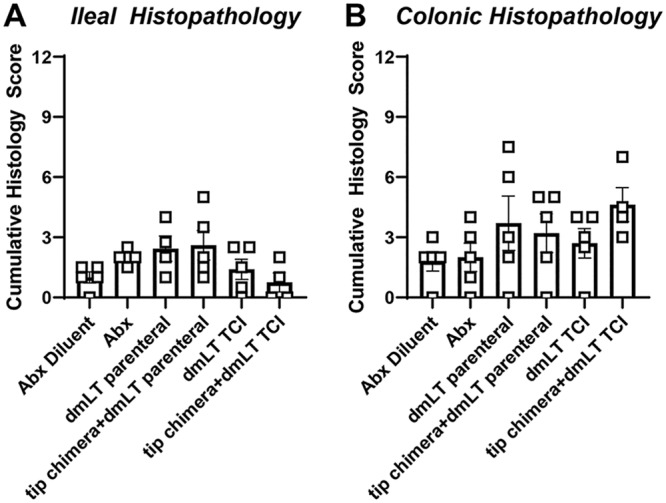
Relative cumulative gastrointestinal tract histopathology among the chinchilla cohorts. Although the mean histological pathology scores were greater in animals that were given antibiotics or immunized with either dmLT or the tip-chimer peptide–dmLT than in animals that received diluent only, these differences were not statistically significant for tissues recovered either from the ileum (ANOVA, *P = *0.145) or from the colon (ANOVA, *P = *0.111).

Neither antibiotic treatment nor immunization with either dmLT or the tip-chimer peptide–dmLT (whether via SQ delivery or TCI) resulted in a significant increase in histological pathology scores; all animals in the study had no to mild histological pathology (Fig. 5). Although the mean histological pathology scores were greater in animals given antibiotics or immunized with either dmLT or the tip-chimer peptide–dmLT than in animals that received diluent only, these differences were not large enough to be considered statistically significantly different from those observed in similar tissues recovered from control cohorts either for the ileum (analysis of variance [ANOVA], *P = *0.145) or for the colon (ANOVA, *P = *0.111) (Fig. 5).

## DISCUSSION

Vaccination is the most cost-effective way to manage infectious diseases (34), and there is constant innovation in the methods by which vaccine candidates are identified, designed, adjuvanted, and delivered in an attempt to customize the induction of the most effective and broadly protective immune response (35, 36). In addition to optimization of vaccine efficacy, similar attention is paid to ensuring that no or limited undesired or off-target side effects occur. Whereas this is often achieved via measurement of adverse events, advances in our understanding and monitoring of vaccines postlicensure have highlighted the potential effects that vaccines might have on the human microbiomes in the form of dysbiosis, in addition to eradication of the targeted pathogen. In the case of diseases like OM, which is typically treated with broad-spectrum antibiotics, this consideration now needs to also include the possibility that antibiotic-driven perturbation of the gut microbiome might also alter the desired immune response to vaccines (37).

In the field of OM research, one such example of an undesired side effect of immunization is how the broad use of pneumococcal conjugate vaccines (PCVs) has changed the microbiology of OM (38, 39). Whereas PCVs have significantly reduced the incidence of invasive pneumococcal disease and have had a measurable effect on acute OM caused by serotypes of S. pneumoniae covered by the vaccine (5, 40–42), there has also been a notable emergence of OM caused by serotypes of S. pneumoniae not covered by the vaccine and also an increase in the incidence of OM caused by pathogens other than one of the usual three predominant otopathogens (43, 44). Moreover, the broad use of PCVs has also resulted in a relative increase in the prevalence of OM due to NTHi. Whereas NTHi has always been the predominant pathogen that causes chronic OM, recurrent OM, and OM for which treatment had failed, it now also ranks as the predominant pathogen that causes acute OM (41). As such, the potential for vaccine-induced and undesirable changes in the microbiome or in the normal microbiology of disease has been highlighted for many laboratories working to develop vaccines against NTHi.

As an issue that we, too, needed to consider, we performed what is, to the best of our knowledge, the first known proof-of-concept study in the chinchilla host, which is the gold standard animal model for OM research (45), to assess the potential for immunization with a biofilm-targeted immunogen to induce alterations to the microbiome, with a particular focus on the gastrointestinal tract.

This is important, given that we targeted the DNABII family of proteins, which are ubiquitous among all eubacteria and not just pathogens, for our therapeutic and vaccine development efforts. At least one allele from this family exists in every bacterium examined to date (19). We and others have shown that this family of proteins is a critical component that maintains the structural integrity of the extracellular matrix of bacterial biofilms. Although the level of DNA sequence homology between family members varies, the immune-protective tip region, with its prevalent proline, is structurally conserved. Indeed, antibodies directed against one member of the DNABII family cross-react with virtually all other members of the family with various avidities (19). This cross-reactivity left open the possibility that benevolent bacterial biofilms could also potentially be affected by vaccination.

Given that the current standard of care for a child with OM is the prescribing of antibiotics (46), which induces transient diarrhea and candidiasis (47, 48), we used delivery of amoxicillin-clavulanate as a positive control with the goal of comparing any antibiotic-induced changes to the gut microbiome with the changes that might follow active immunization with a biofilm-targeted immunogen delivered either systemically or locally/transcutaneously. We found that delivery of amoxicillin-clavulanate orally resulted in significant changes in the chinchilla fecal microbiome, including a decrease in the abundance of *Phascolarctobacterium* and increases in the relative abundances of *Bilophila*, *Bacteroides*, and *Escherichia-Shigella*. Although antibiotic-induced changes in the abundances of *Bilophila* and *Phascolarctobacterium* have not been observed in human hosts, adults treated with amoxicillin-clavulanate have increases in the abundance of *Bacteroides* as well as *Escherichia*, *Shigella*, and other members of the *Enterobacteriaceae* family (49). Indeed, increases in the abundance of *Enterobacteriaceae* are commonly observed after antibiotic treatment and are linked to the enrichment of specific antibiotic resistance genes (49, 50). As a result, therapies for OM that do not result in alterations to the intestinal microbiome, especially an enrichment in the abundance of *Enterobacteriaceae*, would be highly desirable.

In contrast to the altered gut microbiome that we observed in the fecal pellets of animals given a broad-spectrum antibiotic, no similar observation was made in animals immunized with the tip-chimer peptide that was delivered with the powerful adjuvant dmLT, regardless of whether we immunized the animals via the more traditional SQ route or by the transcutaneous route via rubbing of the immunogen onto the skin just behind the ear. Whereas both immunization routes induced immunogen-specific antibody, there were clear differences between the relative serum antibody titers, as expected. Both delivery routes are effective preclinically in chinchilla models whether as a protective vaccine (27, 31–33, 51, 52) or as a therapeutic vaccine (18, 30, 53, 54) is delivered. The latter was demonstrated in animals with preexisting OM, in which robust biofilms were present in the middle ear prior to immunization, yet rapid disease resolution and biofilm eradication were achieved. Whereas we had hypothesized *a priori* that TCI would be far less likely to induce alterations to the gut microbiome due to the character of the immune response that follows local immunization of the postauricular skin (30, 31, 54), there was perhaps a greater possible potential for SQ immunization to induce alterations. Still, given that there is limited transudation of serum antibody onto the surface of intact, noninflamed mucosa, we had hypothesized that neither immunization route was highly likely to induce alterations to the gut microbiome, whereas oral delivery of antibiotics had a great potential to do so. Nevertheless, these hypotheses needed to be tested, and here, we were able to conclude that there was indeed no evidence of an immunization-induced gut microbiome alteration, but there was clear evidence of this due to oral antibiotic delivery. Future experiments will examine additional mucosal surfaces, including the nasopharynges (where the otopathogens originate), to determine if there are even subtle changes in the microbiome as a result of either antibiotic use or immunization.

This concordance between the gut dysbiosis documented in children that receive broad-spectrum antibiotics for the treatment of OM and the alteration of the gut microbiome observed in the chinchilla host lends additional support to the use of this animal model for these and similar studies. Further, the absence of evidence of an altered gut microbiome in the gastrointestinal tract of the chinchilla after delivery of the DNABII-targeted tip-chimer peptide vaccinogen, whether by the systemic or the transcutaneous route, was highly encouraging and supportive of our continued vaccine development efforts.

## MATERIALS AND METHODS

### Chinchillas.

Healthy male and female adult chinchillas (Chinchilla lanigera; mean weight, 644 g) were purchased from Rauscher’s Chinchilla Ranch, LLC (LaRue, OH), and rested in a vivarium for 2 weeks to permit acclimatization to the environment as well as to the Mazuri chinchilla diet (catalog number 0001471; PMI Nutrition International LLC) and water sources. All chinchillas were housed on autoclaved corn cob bedding, which was changed once a week, and provided fresh timothy hay, which was replenished weekly. Autoclaved water was provided *ad libitum*, with the bottles being changed every 2 days. Six cohorts of 5 chinchillas each, based on equivalent mean body weight, were established and either given antibiotic or immunized via one of two routes (see Table S1 in the supplemental material) and at the times on the timeline presented in Fig. S1. Chinchilla studies were performed in accordance with federal, state, and institutional guidelines under protocol number 01304AR, which was approved by the Nationwide Children’s Hospital Institutional Animal Care and Use Committee.

10.1128/mSphere.00296-20.3 TABLE S1 Dose, route, and vaccine formulation or antibiotic administered to cohorts of chinchillas. Download Table S1, PDF file, 0.1 MB. Copyright © 2020 Bailey et al. 2020 Bailey et al. This content is distributed under the terms of the Creative Commons Attribution 4.0 International license.

### Antibiotic or immunogen delivered.

Amoxicillin and clavulanate potassium (catalog number NDC 65862-0533-01; Aurobindo Pharma) was administered to one cohort to represent the standard recommended first-line treatment of children with acute OM (46). The powder was suspended in sterile, pyrogen-free water and stored at 4°C. Sterile, pyrogen-free water served as the negative control for antibiotic delivery.

The tip-chimer peptide incorporates 20-mer immunoprotective domains from within the DNA-binding tips of both the alpha subunit and the beta subunit of the DNABII protein integration host factor (IHF) expressed by nontypeable Haemophilus influenzae (NTHi) strain 86-028NP joined by a 4-mer linker peptide (27, 55). Vaccine formulations consisted of 10 μg tip-chimer peptide admixed with 10 μg of the adjuvant LT (R192G/L211A), a double mutant of Escherichia coli heat-labile enterotoxin (dmLT; a generous gift from John D. Clements, Tulane University) (56). Delivery of 10 μg dmLT only served as the negative control.

### Administration of antibiotic or immunization regimens.

Ten milligrams of amoxicillin-clavulanate per kilogram of body weight was delivered orally to alert animals in a divided dose twice a day. An equivalent volume of diluent (sterile, pyrogen-free water) served as a negative control (Fig. S1). Whereas a 10-day course of amoxicillin-clavulanate was intended to mimic the standard of care for children with OM, signs of animal distress (significantly decreased gastrointestinal motility, altered character of fecal pellets, and cornering behavior) were observed, and we therefore ceased treatment after 7 days. Animals in the cohorts administered antibiotic or diluent were humanly euthanized on day 8.

Transcutaneous immunization with tip-chimer peptide–dmLT or dmLT alone was performed as a modification of a previously described technique (30). At 1 day prior to immunization, the fur directly caudal to each chinchilla outer ear (the postauricular region) was plucked and the animals rested for an additional 24 h to permit resolution of any resultant nonspecific inflammation. Alert animals were then immunized by rubbing vaccine formulations onto the intact and unabraded postauricular skin; a total of 5 μg tip-chimer peptide–5 μg dmLT or 5 μg dmLT was applied, such that a total immunizing dose of 10 μg tip-chimer peptide–10 μg dmLT or 10 μg dmLT only was administered. This procedure was repeated 8 days later (Fig. S1).

Parenteral immunization was performed by subcutaneous injection of 100 μl of tip-chimer peptide–dmLT or dmLT alone along the rear flank of each chinchilla (32). A total immunizing dose of 10 μg tip-chimer peptide–10 μg dmLT or 10 μg dmLT only was administered to each animal on study days 0, 30, and 60 (Fig. S1).

### Fecal pellet collection.

Alert animals were placed individually into clean, empty cages, and a single fecal pellet was collected upon evacuation, immediately transferred into sterile microcentrifuge tubes using sterile forceps, and snap-frozen over the vapor phase of liquid nitrogen, in which it was also stored until processed. The fecal pellets collected from all animals after a 14-day acclimation period are referred to as “day 0” or “baseline” samples. The time points for additional fecal pellet collection and the rationale associated with their collection at those time points are described below.

Fecal pellets were also collected from cohorts administered antibiotic or diluent on day 8 (Fig. S1). Fecal pellets were collected from animals immunized by TCI with tip-chimer peptide–dmLT or dmLT only on study day 8 (to permit direct comparison to the Abx-treated cohort and to examine potential changes induced after only one dose) and upon regimen completion on day 17. Fecal pellets were collected from the cohort parenterally immunized with tip-chimer peptide–dmLT or dmLT alone on study days 8 and 17 (to permit direct comparison with the antibiotic-treated and TCI cohorts), in addition to day 30 (which was the midpoint of the entire 60-day immunization regimen, to examine potential changes induced after only one immunization) and upon regimen completion on day 70 (the time point at which high-titer antisera were detected).

### Collection of blood and assay of serum antibodies by ELISA.

To retrieve blood from the immunized cohorts by cardiac puncture, the animals were first anesthetized by intramuscular injection of 10 mg ketamine and 2 mg xylazine per kg of body weight. Blood was stored at 4°C for 24 h to permit clotting and then centrifuged at 1,800 × *g* at 4°C for 30 min to collect serum, which was then stored at −80°C until it was assayed. To determine the reciprocal titer of tip-chimer-specific antibody, an endpoint enzyme-linked immunosorbent assay (ELISA) was performed as previously described (54). Briefly, sera were incubated in tip-chimer peptide-coated wells (0.2 μg peptide/well) for 1 h at 25°C, and bound antibody was detected with horseradish peroxidase-conjugated protein A (catalog number 101023; Invitrogen). Color was developed with 3,3′,5,5′-tetramethylbenzidine (TMB; catalog number 34028; Pierce Biotechnology). Endpoint reciprocal titers were defined as the dilution that yielded an optical density at 450 nm value of 0.1 above that value for control wells, which were incubated without serum, and are reported as the geometric mean titer (GMT) ± standard deviation, as determined with GraphPad Prism (version 8) software.

### Measured outcomes.

(i) A longitudinal assessment of whether immunization with the DNABII-directed candidate induced alterations in the gut microbiome from the gut microbiome seen in the respective control cohort administered adjuvant only and the cohort administered antibiotic was performed. Whereas we had originally intended to also assess for alterations in the airway microbiome via collection of NP lavage fluids, as stated earlier, due to the low sequencing depth and the high variability in the taxa found in these samples, we had limited confidence in the data and thereby did not ultimately subject them to further analyses. (ii) The gastrointestinal tract mucosae were examined via histology to ascertain changes in the architecture of the mucosal epithelium, including disruption of the barrier function, goblet cell hyper- or hypoplasia, villus height, sloughing of epithelial cells, or the presence of red blood cells by hematoxylin and eosin staining of midcolon tissue sections.

### Histological scoring.

Upon sacrifice, a portion of tissue within the distal ileum and the midpoint of the colon was collected from each animal for histological analysis. A transverse section of the ileum and the midcolon was fixed in methanol-Carnoy solution, embedded in paraffin, sectioned to a 5-μm thickness, and stained with hematoxylin and eosin. Two independent investigators blind to the treatments scored the sections based on four criteria: inflammatory cell infiltrates, epithelial/crypt disruptions, goblet cell depletion, and submucosal edema (57). Each criterion was scored on a scale of from 0 to 3, based on the following: for inflammatory cell infiltrates, no infiltrate was given a score of 0, an increased presence of inflammatory cells in the lamina propria only was given a score of 1, an increased presence of inflammatory cells in the lamina propria and the submucosa was given a score of 2, and the presence of transmural inflammatory infiltrates was given a score of 3; for epithelial/crypt disruptions, no disruption was given a score of 0, mild disruption was given a score of 1, moderate disruption was given a score of 2, and severe disruption was given a score of 3; for goblet cell depletion, no depletion was given a score of 0, 10% depletion was given a score of 1, 11 to 50% depletion was given a score of 2, and >50% depletion was given a score of 3; and for submucosal edema, no edema was given a score of 0, mild edema was given a score of 1, moderate edema was given a score of 2, and severe edema was given a score of 3. The scores of the four criteria were summed for a total maximum score of 12.

### 16S rRNA gene sequencing.

DNA was extracted from the NP lavage fluid (250 μl) or fecal pellets (∼10 mg) using a QIAamp Fast DNA stool minikit (catalog number 51604; Qiagen) following the manufacturer’s instructions, with slight modifications. Briefly, samples were incubated for 45 min at 37°C in lysozyme buffer (22 mg lysozyme/ml, 20 mM Tris-HCl, 2 mM EDTA, 1.2% Triton X-100, pH 8.0) and then bead beaten for 150 s with 0.1-mm zirconia beads. The samples were incubated at 95°C for 5 min with InhibitEX buffer and then incubated at 70°C for 10 min with proteinase K and buffer AL (catalog number 19075; Qiagen). Following this step, the QIAamp Fast DNA stool minikit isolation protocol was followed, beginning with the ethanol step. DNA was quantified with a Qubit (version 2.0) fluorometer using a double-stranded DNA broad-range assay kit (catalog number Q32853; Invitrogen). Samples were submitted to the Argonne National Laboratory (Lemont, IL) for library preparation and amplicon sequencing. Amplified PCR libraries were sequenced from both ends of the 250-nucleotide region of the V4-V5 16S rRNA hypervariable region using an Illumina MiSeq sequencer (San Diego, CA). The DADA2 and Quantitative Insights into Microbial Ecology (QIIME; version 2.0) programs were utilized for amplicon processing, quality control, and downstream taxonomic assignment using the rRNA database SILVA (release 137).

### Statistical analyses.

Bacterial community alpha diversity was measured using the Shannon diversity index. Welch’s *t* test was used to compare groups on specific days of treatment, whereas paired-samples *t* tests were used to compare the Shannon diversity indexes at specific days posttreatment to the study day 0 values. Bacterial community beta diversity was measured using Bray-Curtis dissimilarities and assessed using PerMANOVA with 999 randomizations of the data. When bacterial community beta diversity was found to differ between two treatments, random forest (RF) classification was utilized to identify bacteria that were differentially abundant in the two treatments. Random forest analysis was performed on the relative abundances of the bacterial genera using at least 1,000 trees, with the Matthews correlation coefficient (MCC) used as an assessment for classification efficacy (58, 59). Boruta feature selection was used to understand which microbes best discriminated between antibiotic treatment and control conditions (60). Boruta feature selection uses RF analysis and iteratively compares the importance of variables with the importance of pseudorandom variables. Variables that have significantly worse importance than the pseudorandom variables are consecutively dropped, thereby limiting the chance of random significance and type I errors (59). Variables that were confirmed by Boruta selection were deemed to be different between the treatment and the control conditions.
