# Supplementary figures and images for: Immunization with a Biofilm-Disrupting Nontypeable Haemophilus influenzae Vaccine Antigen Did Not Alter the Gut Microbiome in Chinchillas, Unlike Oral Delivery of a Broad-Spectrum Antibiotic Commonly Used for Otitis Media
Source: mSphere. 2020 Apr 15;5(2):e00296-20. doi: 10.1128/mSphere.00296-20 (PMC7160684; doi:10.1128/mSphere.00296-20)

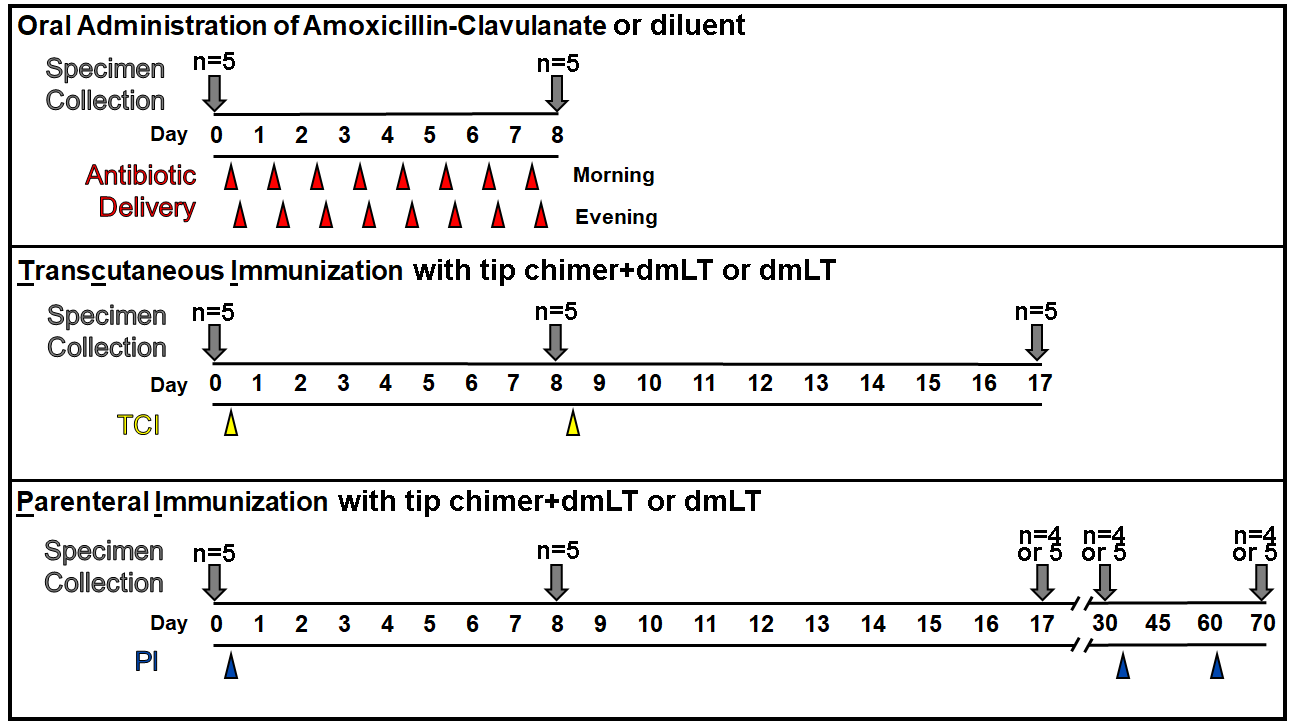

Supplement: FIG S1 [file mSphere.00296-20-sf001.tif]

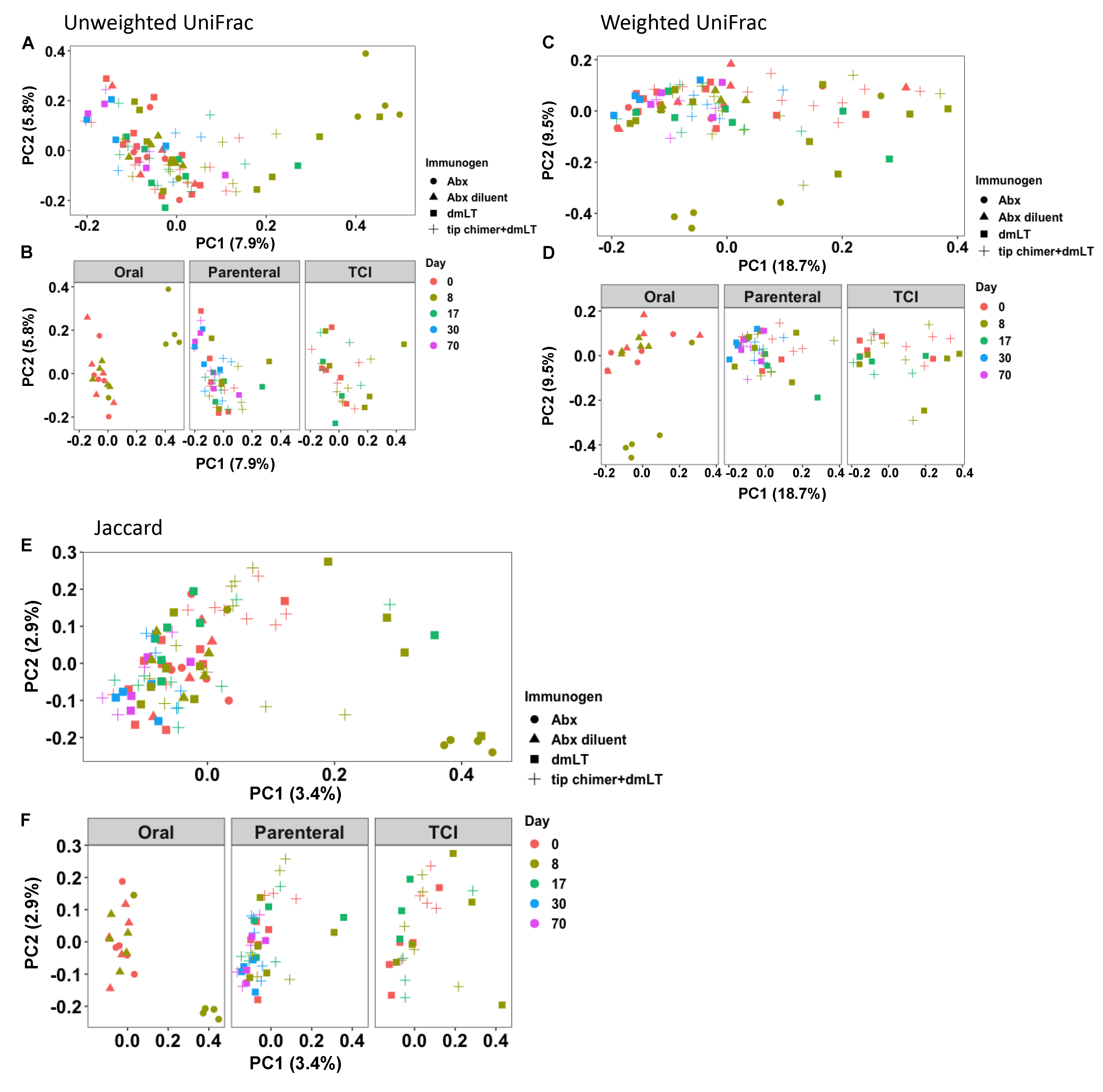

Supplement: FIG S2 [file mSphere.00296-20-sf002.tif]
