# Supplementary material for: Immunization with a Biofilm-Disrupting Nontypeable Haemophilus influenzae Vaccine Antigen Did Not Alter the Gut Microbiome in Chinchillas, Unlike Oral Delivery of a Broad-Spectrum Antibiotic Commonly Used for Otitis Media
Source: mSphere. 2020 Apr 15;5(2):e00296-20. doi: 10.1128/mSphere.00296-20 (PMC7160684; doi:10.1128/mSphere.00296-20)
Supplement: TABLE S1 [file mSphere.00296-20-st001.pdf]

<u>Supplemental Table 1</u>. Dose, route and vaccine formulation or antibiotic administered to cohorts of chinchillas.

Antibiotic/ vaccine formulation administered	Delivery route	Dose	Volume administered
Amoxicillin-clavulanate	Oral delivery[a]	10 mg/ kg/ day	80 µl
sterile water	Oral delivery[a]	Not applicable	80 µl
IHF_{NTHI} tip chimer peptide + dmLT	Transcutaneous immunization[b]	10 µg peptide + 10 µg adjuvant	100 µl
dmLT	Transcutaneous immunization[b]	10 µg adjuvant	100 µl
IHF_{NTHI} tip chimer peptide + dmLT	Parenteral immunization[c]	10 µg peptide + 10 µg adjuvant	100 µl
dmLT	Parenteral immunization[c]	10 µg adjuvant	100 µl

[a]Antibiotic solution or diluent pipetted directly into the oral cavity

[b]Formulation rubbed on the skin at the post-auricular region, 50 µl applied at each site

[c]Formulation injected subcutaneously along rear flanks
